# Effects of galacto-oligosaccharides on growth and gut function of newborn suckling piglets

**DOI:** 10.1186/s40104-018-0290-9

**Published:** 2018-10-18

**Authors:** Shiyi Tian, Jue Wang, Hu Yu, Jing Wang, Weiyun Zhu

**Affiliations:** National Center for International Research on Animal Gut Nutrition, Jiangsu Key Laboratory of Gastrointestinal Nutrition and Animal Health, Laboratory of Gastrointestinal Microbiology, College of Animal Science and Technology, Nanjing Agricultural University, Nanjing, 210095 China

**Keywords:** Early intervention, Galacto-oligosaccharides, Growth performance, Intestinal development, Jejunum, Suckling piglets

## Abstract

**Background:**

Most research on galacto-oligosaccharides (GOS) has mainly focused on their prebiotic effects on the hindgut, but their beneficial effects on the small intestine (SI) have received little attention. Since jejunum is the important place to digest and absorb nutrients efficiently, optimal maturation of the jejunum is necessary for maintaining the high growth rate in the neonate. Therefore, this study investigates the effect of the early intervention with GOS on the intestinal development of the jejunum.

**Methods:**

A total of 6 litters of neonatal piglets (10 piglets per litter; Duroc × Landrace × Large White) with an average birth weight of 1.55 ± 0.05 kg received 1 of 2 treatments based on their assignment to either the control (CON) group or the GOS (GOS) group in each litter. Piglets in the GOS group were orally administrated 10 mL of a GOS solution (reaching 1 g GOS/kg body weight) per day from the age of 1 to 7 d; the piglets in the CON group were treated with the same dose of physiological saline. All piglets were weaned on d 21. On d 8 and 21 of the experimental trial, 1 pig per group from each of the 6 litters was euthanized.

**Results:**

The early intervention with GOS increased the average daily gains in the third week (*P* < 0.05). Decreased crypt depth was also observed in the jejunum of the piglets on d 21 (*P* < 0.05). The early intervention with GOS increased the jejunal lactase activity on d 8, maltase activity and sucrase activity on d 21 (*P* < 0.05). In addition, the early intervention with GOS also facilitated the mRNA expression of Sodium glucose co-transporter 1 (*SGLT1*) on d 8 and the mRNA expression of Glucose transporter type 2 (*GLUT2*) on d 21 (*P* < 0.05). It was further determined that GOS up-regulated the mRNA expression of preproglucagon (*GCG*), insulin-like growth factor 1 (*IGF-1*), insulin-like growth factor 1 receptor (*IGF-1R*) and epidermal growth factor (*EGF*). GOS also up-regulated the protein expression of glucagon-like peptide-2 (GLP-2) and EGF in the jejunum of the piglets. Furthermore, it was also found that GOS enhanced the protein expression of ZO-1 and occludin on d 8 (*P* < 0.05), as well as increased the mRNA expression of *TGF-β* and decrease the mRNA expression of *IL-12* (*P* < 0.05).

**Conclusions:**

These results indicate that GOS have a positive effect on piglet growth performance in addition to decreasing the crypt depth and enhancing functional development in jejunum of suckling piglets.

**Electronic supplementary material:**

The online version of this article (10.1186/s40104-018-0290-9) contains supplementary material, which is available to authorized users.

## Background

A nutritional strategy to improve the growth performance of newborn animals is the basis of improving the overall productivity of animals [1]. The jejunum is an important part of the intestine in which the efficient digestion and absorption of nutrients take place, and the maturation of the jejunum is therefore beneficial for maintaining a high rate of growth among neonates [2, 3]. During the neonatal period, the gastrointestinal tract of piglets develops rapidly [4]. Dietary nutrients are essential for gastrointestinal (GI) growth and functional development, and the nutritional support of GI growth and development is a significant part of the nursing process [5].

In recent years, probiotics and prebiotics have gained considerable attention as growth promoters. Galacto-oligosaccharides (GOS), a type of prebiotics, contain 2-8 saccharide units, where one of these units is terminal glucose and the remaining are galactoses and disaccharides comprised of 2 units of galactoses [6, 7]. GOS are attractive food additives for infant formula because of their capability of modulating the intestinal microbiota, improving intestinal development, enhancing mineral absorption, and protecting the intestinal barrier [8, 9]. Previous studies have shown that GOS could promote the growth of beneficial bacteria and improve host health in vitro and in vivo [10–12]. However, the promotion of intestinal development and the enhancement of intestinal barrier properties by GOS have been described mainly in vitro using cell models such as the human Caco-2 cell line and rodent models [13, 14]; very little has been shown about the effects on suckling piglets in vivo. Moreover, most in vivo studies of GOS have been specifically restricted to the cecum and colon but very few data are currently available for the jejunum. It is essential to explore the effect of early intervention with GOS on the jejunal development of suckling piglets.

We hypothesized that early intervention with GOS could increase the growth performance and improve the intestinal development of suckling piglets. The present study was conducted to explore the effects of early intervention with orally administrated GOS on the jejunal morphology, digestive and absorptive functions, and barrier functions of suckling piglets.

## Methods

### Animals and experimental treatments

A total of 6 litters of neonatal piglets (10 piglets in each; Duroc × Landrace × Large White) were used in this study with an average birth weight of 1.55 ± 0.05 kg. Piglets from each litter were equally assigned to either the control (CON) group or the GOS (GOS) group to receive 1 of 2 treatments in order to account for any maternal differences. GOS-90S was obtained from Quantum Hi-Tech Biological Co., Ltd. (China), which contained oligosaccharides with a degree of polymerization (DP) of 2–8 with approximately 90% (*w*/*w*) GOS, 8.5% (*w/w*) lactose, and 1.5% (*w/w*) glucose on dry matter (DM). During the 7 d after birth, all piglets in the GOS group were orally administered 10 mL galacto-oligosaccharides (GOS) solution (1 g GOS/kg body weight [9, 10, 15, 16]) per day. Half of the dose was given at 9:00 and the other at 18:00. Similarly, all the piglets in the CON group were orally administered the same dose of physiological saline. The solution was infused into each piglet’s mouth by an injector without a needle with a soft infusion tube with a length of 5 cm. To avoid the potential stress after the swallowing, the piglets were put in the nursing pen immediately. All piglets were weaned on d 21. The piglets had free access to sow milk and water at all times throughout the experimental period. Average bodyweight throughout the treatment process was recorded on d 7, 14 and 21. Health status was monitored daily until 21 d of age, and all piglets remained healthy during the experimental period.

### Sample collection and measurement of villus morphology

On d 8 and 21 of the experimental trial, 1 pig per group from each of the 6 litters was euthanized with an intravenous overdose of pentobarbital via a catheterized ear vein as described by Moeser et al. [17]. Blood samples were collected from the anterior vena cava into tubes containing sodium heparin and immediately mixed to avoid coagulation. Plasma was obtained after centrifugation at 3,000×*g* for 15 min at 4 °C and then stored at − 80 °C until analysis. The small intestine (SI) was also removed. The length of the SI was measured after stripping off the mesentery by holding the intestine vertically against a ruler. The wet weight of the SI was determined after gently squeezing out the intestinal contents and removing the mesentery and fat.

The mid-jejunal samples (midpoint between the pylorus and the ileocecal valve) were preserved in a formaldehyde and glutaraldehyde mixing fixative. Then the cross-sections of mid-jejunal samples were prepared using standard paraffin embedding techniques. To measure villus height and crypt depth, the samples were sectioned at 6 μm thickness and stained with hematoxylin and eosin (HE) [18]. Mucosal samples from the proximal jejunum (at 40 cm from the duodenum-jejunum junction) were collected using a glass slide, rapidly frozen in liquid nitrogen, and stored at − 80 °C for further analysis. All samples were collected within 20 min after the piglet had been euthanized. The total protein in the mucosal was extracted according to the instructions of a total protein extraction kit (KGP2100; Keygen Biotech, Nanjing, China). The mucosal protein concentration of the supernatant fractions was then quantified by a standard bicinchoninic acid (BCA) protein assay (A045–3, JianCheng Bioengineering Institute, Nanjing, Jiangsu, China).

### RNA extraction, cDNA synthesis, and real-time RT-PCR for gene expression analysis

The frozen jejunal mucosa was homogenized in 1 mL Trizol Reagent (Invitrogen, Carlsbad, CA, USA), and the total RNA was isolated according to the manufacture’s recommendations. The absorption ratio (260/280 nm) of all the samples was between 1.8 and 2.0, which indicated high purity of the RNA. The total RNA was reverse-transcribed to cDNA using a PrimeScript RT reagent kit with a gDNA eraser (Takara Biotechnology (Dalian) Co., Ltd.) according to the recommended procedures.

The primers for the intestinal nutrient transporter genes: sodium glucose co-transporter 1 (*SGLT1*), glucose transporter type 2 (*GLUT2*); the intestinal growth factors: preproglucagon (*GCG*), insulin-like growth factor 1 (*IGF-1*), insulin-like growth factor 1 receptor (*IGF-1R*), epidermal growth factor (*EGF*); the intestinal barrier related genes: zonula occludens-1 (*ZO-1*), occludin; the intestinal immune factors: interleukin-1β (*IL-1β*), interleukin-10 (*IL-10*), interleukin-12 (*IL-12*), toll-like receptor 4 (*TLR4*), transforming growth factor-β (*TGF-β*), tumor necrosis factor-α (*TNF-α*) and housekeeping genes (*β-actin* and glyceraldehyde phosphate dehydrogenase (*GAPDH*)) are listed in Additional file 1: Table S1.

The target genes and housekeeping genes were measured with an Applied Biosystems 7300 Real-Time PCR system using a SYBR Premix Ex Taq™ (Tli RnaseH Plus) qPCR kit (Takara Biotechnology (Dalian) Co., Ltd.) according to the manufacturer’s guidelines. The standard dilution and samples were assayed in triplicate in a 20 μL reaction mixture containing 10 μL of SYBR, 0.4 μL 0.2 μmol/L of forward and reverse primer, 6.8 μL nuclease-free water, and 2 μL of 100 ng/μL DNA template. The cycling conditions were 95 °C for 30 s, followed by 40 cycles of 95 °C for 5 s and 60 °C for 34 s. The standard curve was also included in each run to determine PCR efficiency. The specificity and efficiency of the selected primers were confirmed by qRT-PCR analysis and a dilution series of pooled cDNA at a temperature gradient (55–65 °C) for primer-annealing and subsequent melting curve analysis. The stability of the housekeeping genes was evaluated by measuring the fluctuation range of the Ct values (Ct values were obtained by real-time quantitative PCR). Then, the two candidate genes were analyzed by NormFinder software [19]. The *β-actin* was finally identified as the housekeeping gene because no variation in its expression was observed between treatments. The mRNA expression levels were calculated using the 2^-ΔΔCt^ method [20]. All the data were normalized to those of the housekeeping gene *β-actin*. The CON group was then established as the control group. The relative expression of the target gene mRNA in each group was calculated as follows: ΔCt = Ct (target gene) - Ct(*β-actin*), and ΔΔCt = ΔCt (treated group) - ΔCt (CON group on d 8).

### *D*-lactic acid and diamine oxidase

The levels of diamine oxidase (DAO; Enzyme Commission Number (EC) 1.4.3.6) and *D*-lactic acid were used as the indices of intestinal mucosal injury in the piglets. The levels of *D*-lactic acid in the plasma were determined with a *D*-lactic acid colorimetric assay kit (BioVision Inc., Milpitas, CA). *D*-lactic acid in the plasma was expressed in terms of mg/L. Diamine oxidase activities in the plasma and jejunal mucosa were measured using an enzymatic spectrophotometric assay as described by Hu et al. [21]. Diamine oxidase activities in the plasma and jejunal mucosa were expressed in terms of units/mL and units/mg mucosa, respectively.

### Disaccharidase activity

The enzymes studied here were lactase (EC 3.2.1.23), sucrase (EC 3.2.1.48) and maltase (EC 3.2.1.20). All assays were carried out on the homogenates of mucosal tissue obtained by thawing approximately 0.1 g of tissue and homogenizing it in 9 mL phosphate buffer saline (PBS, pH = 7.2) with an ultrasonic homogenizer. The homogenate was then centrifuged (500×*g*, 10 min at 4 °C), and the supernatants were collected. The activity levels of the digestive enzymes lactase (Lactase Activity Testing Kit, No: A082–1), sucrase (Sucrase Activity Testing Kit, No: A082–2) and maltase (Maltase Activity Testing Kit, No: A082–3) were determined according to the instructions of the manufacturer Nanjing JianCheng Bioengineering Institute (Nanjing, Jiangsu, China).

### Intestinal growth factors

The levels of intestinal growth factors (glucagon-like peptide-1 (GLP-1), glucagon-like peptide-2 (GLP-2), insulin-like growth factor 1 (IGF-1), and epidermal growth factor (EGF)) in the intestinal mucosa were determined using the ProcartaPlex™ multiplex immunoassay kit (Luminex, Austin, USA) according to the manufacturer’s instructions obtained from Affymetrix eBioscience (Santa Clara, USA). The results were normalized against the total protein concentration for each sample in an inter-sample comparison.

### Tight junction protein expressions

Tight junction protein expressions of zonula occludens-1 (ZO-1) and occludin were measured by Western blotting. After the protein concentration of supernatant fractions was quantified by a standard bicinchoninic acid (BCA) protein assay (Pierce, Rockford, IL, USA), the standardized protein amounts of boiled samples were isolated with a 15% sodium dodecyl sulfate–polyacrylamide gel electrophoresis (SDS–PAGE) and electro-transferred onto polyvinylidene difluoride (PVDF) membranes (Merck Millipore). Membranes were blocked in a skim milk TBS buffer (15 mmol/L Tris-HCl, 150 mmol/L NaCl, 10% skim milk; pH 7.4) and incubated overnight at 4 °C with antibodies for occludin (1:1,000; Abcam) or ZO-1 (1:1,000; Invitrogen). After being washed in a phosphate buffer solution with Tween-20 (PBST), the membranes were incubated with an appropriate horseradish peroxidase-conjugated secondary antibody (1: 2,000; Fcmacs-Bio, Beijing, China) for 2 h at room temperature. Finally, the PVDF membranes harboring the target bands were visualized through an electrochemiluminescence system (Tanon, Shanghai, China). Subsequently, the membranes were re-probed with a β-actin antibody (1:2,000; Cell Signaling) to assess the equality of loading. Band intensities were quantified using ImageJ version 1.47 software (National Institute of Health, American), and the protein expression was normalized with β-actin and expressed as the mean fold change in relation to the control group.

### Statistical analysis

Data were analyzed by SPSS 20.0 (IBM, US) and expressed as means ± SEM or means ± SD. The model included the fixed effects of diet, age, associated interactions, and any random errors with respect to a group or an individual piglet. Bodyweight (BW) and average daily gain (ADG) were evaluated using the group in each litter as the experimental unit. The other parameters were assessed using each slaughtered piglet as an experimental unit. The data were evaluated by two-way ANOVA, and differences were considered significant at *P* < 0.05. When a significant dietary effect or an interaction between diet and time was observed, the data were further analyzed by using one-way ANOVA with Duncan’s post hoc test. And a value of *P* < 0.05 was used to indicate statistical significance, whereas a *P*-value between 0.05 and 0.10 was considered to indicate a trend toward significance.

## Results

### Growth performance and digestive organ indexes

**Fig. 1:**
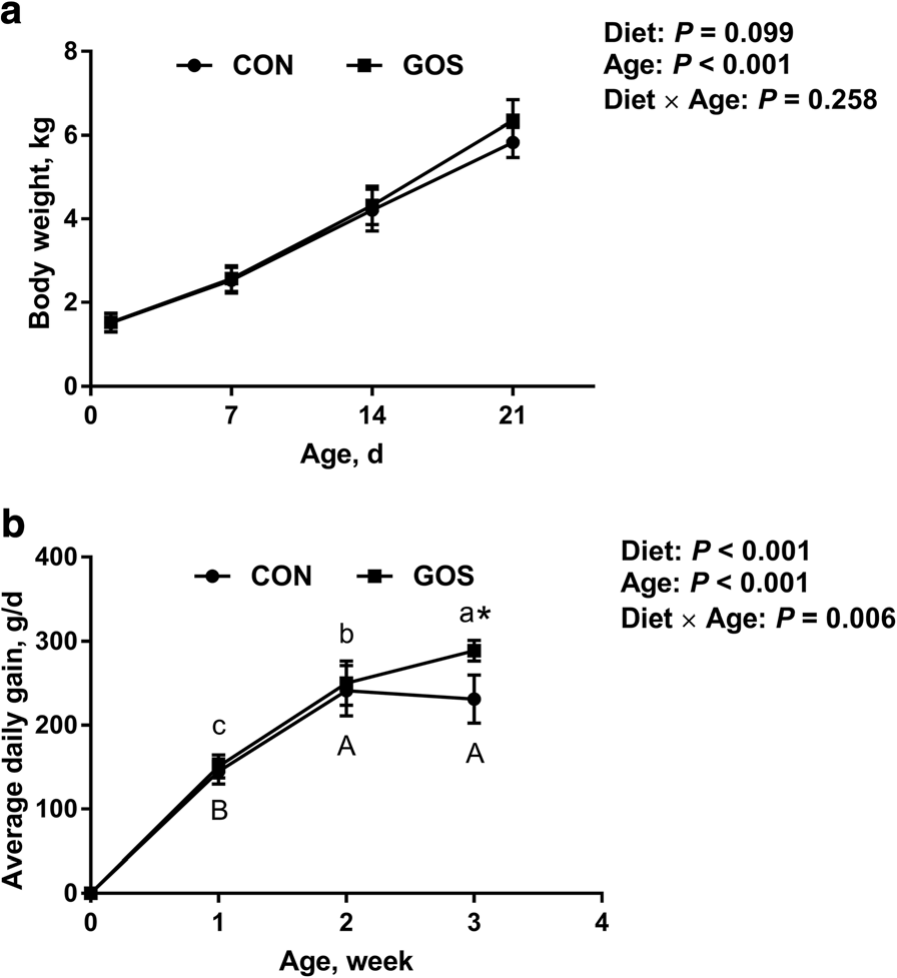
Effects of galacto-oligosaccharides (GOS) on growth performance of suckling piglets. Piglets assigned to CON (*n* = 6) and GOS (*n* = 6) received physiological saline and GOS solution for 7 d after birth, respectively. (**a)** Body weight and (**b**) Average daily gain of suckling piglets. Significant differences (*P* < 0.05) among different aged piglets within each diet group are indicated by different letters (upper case for the CON group, lower case for GOS group). *Indicates a significant difference (*P* < 0.05) between the diets at each age. Values are expressed as means ± SD, CON: control group; GOS: GOS group

The effects of early intervention with GOS on the growth performance of suckling piglets are illustrated in Fig. 1. BW was significantly (*P* < 0.05) affected by the age of piglets, and it also tended to be affected by diet (*P* = 0.099). Piglets fed with diets containing GOS showed higher BW than those fed with no GOS on d 21, but the difference was not significant. For ADG, there was a significant interaction (*P* < 0.05) between diet and the age of the piglets. Piglets fed with diets containing GOS showed significantly higher ADG (*P* < 0.05) than those fed with no GOS in the third week. In addition, the results with respect to the digestive organs are presented in Table 1. SI length was significantly affected by diet (*P* < 0.05), and the same tendency was reported for SI weight (*P* = 0.078); SI weight / BW tended to be affected by the interaction between diet and age (*P* = 0.096). Furthermore, piglets fed with diets containing GOS showed significantly increased SI length (*P* < 0.05) than those fed with no GOS on d 8.

**Table 1:** Effects of galacto-oligosaccharides (GOS) on digestive organ indexes of suckling piglets^a^

Items	d 8		d 21		SEM	*P*-value		
	CON	GOS	CON	GOS		Diet	Age	Diet × Age
SI^b^ weight, g	79.72	107.32	193.23	197.74	8.66	0.078	< 0.001	0.196
SI length, m	4.39 ^c^	4.93 ^b^	6.64 ^a^	6.81 ^a^	0.17	0.041	< 0.001	0.290
SI weight/SI length, g/m	19.03	21.50	29.06	28.86	0.82	0.183	< 0.001	0.121
SI weight/BW, g/kg	26.85	31.92	31.45	31.20	1.53	0.131	0.218	0.096
SI length/BW, m/kg	1.43	1.49	1.08	1.08	0.04	0.553	< 0.001	0.473

^a^Piglets assigned to CON (*n* = 6), GOS (*n* = 6) received physiological saline and GOS solution for 7 d after birth, respectively

^b^*SI* small intestinal

### Intestinal morphology and intestinal growth factors

**Fig. 2:**
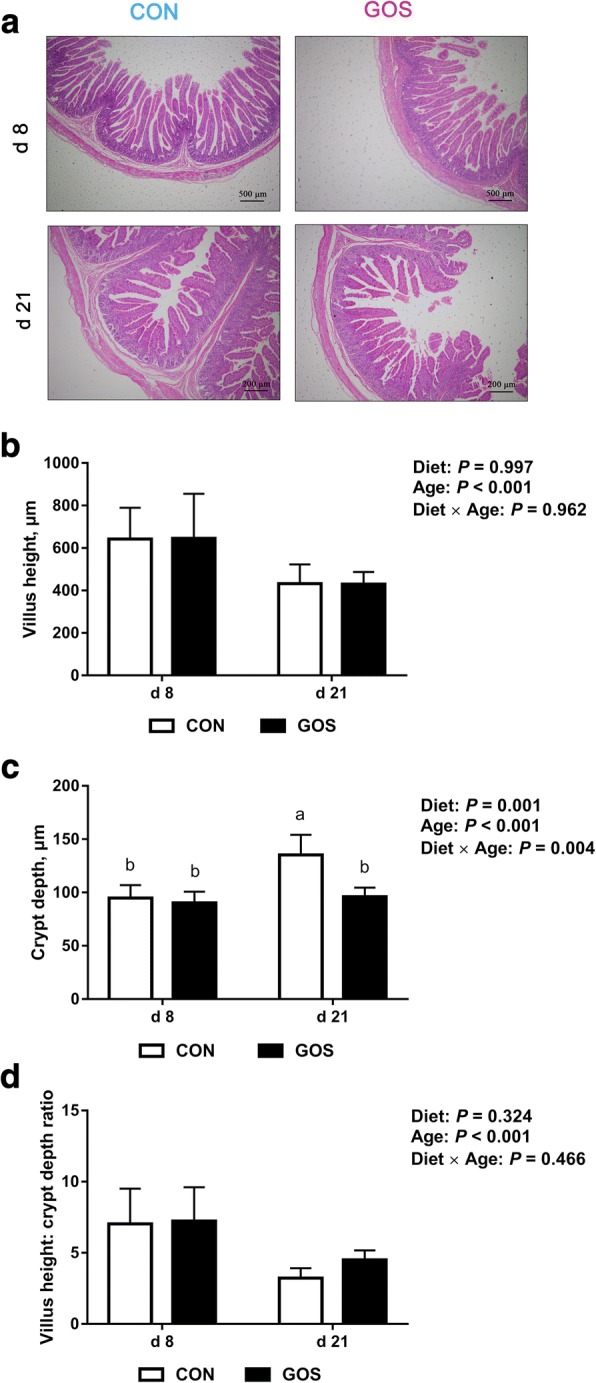
The jejunal morphology of suckling piglets. Piglets assigned to CON (*n* = 6) and GOS (*n* = 6) received physiological saline and GOS solution for 7 d after birth, respectively. (**a)** Representative histological micrographs of jejunum in suckling piglets. The scale bar of jejunal morphology on d 8 was 500 μm, and the scale bar of jejunal morphology on d 21 was 200 μm. (**b**) Villus height, (**c**) Crypt depth and (**d**) Villus height: crypt depth radio of jejunal morphology in suckling piglets. Values are expressed as means ± SD. Bars assigned with different lower-case letters indicate a significant difference. CON: control group; GOS: GOS group

Diet and age had no significant interactive effects on villus height and the villus height / crypt depth ratio of the jejunum of piglets (*P* > 0.05). Crypt depth was significantly affected by the interaction between diet and age (*P* < 0.05). Piglets fed with diets containing GOS showed significantly less crypt depth (*P* < 0.05) than those fed with no GOS on d 21 (Fig. 2).

**Fig. 3:**
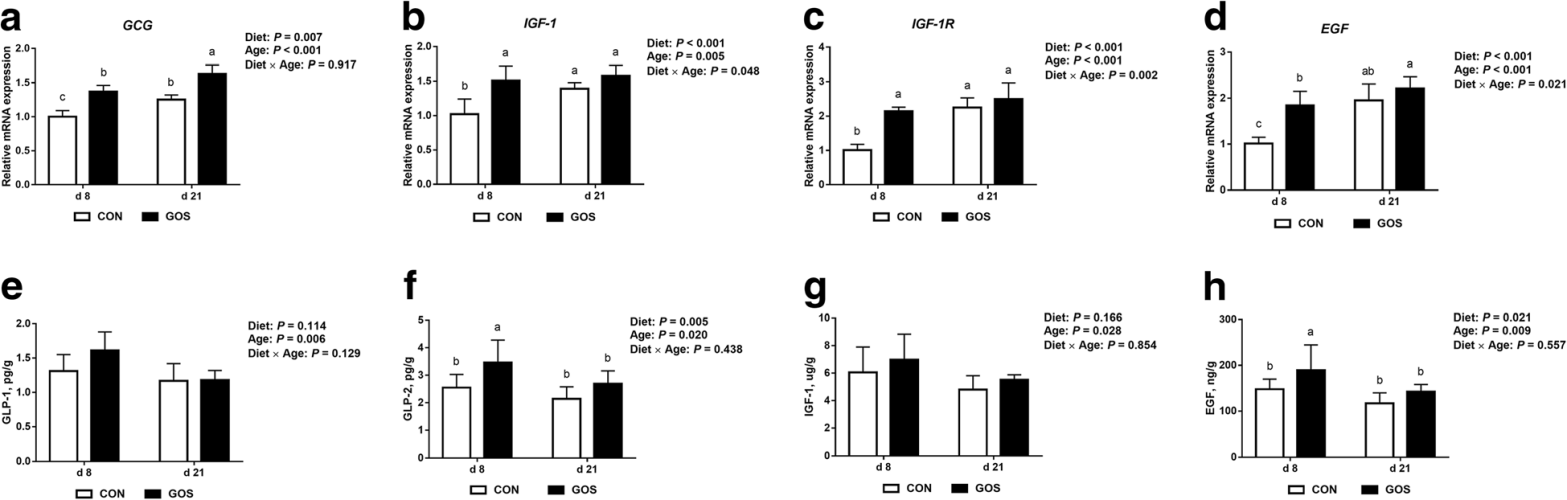
The expression of jejunal growth factors in suckling piglets. Piglets assigned to CON (*n* = 6) and GOS (*n* = 6) received physiological saline and GOS solution for 7 d after birth, respectively. **a**-**d** The relative mRNA expression of jejunal growth factors in suckling piglets. The values were calculated relative to the expression of *β-actin* with formula 2^-∆∆Ct^. **e**-**h** The concentrations of jejunal growth factors in suckling piglets. Values are expressed as means ± SD. Bars assigned with different lower-case letters indicate significant differences. CON: control group; GOS: GOS group

The effects of early intervention with GOS on the mRNA expression of intestinal growth factors are presented in Fig. 3a-d. The mRNA expression of *IGF-1*, *IGF-1R*, and *EGF* was affected by interactions between diet and age (*P* < 0.05), and the mRNA expression of *GCG* was affected by diet. Piglets fed with diets containing GOS showed significantly higher mRNA expression of *GCG*, *IGF-1*, *IGF-1R* and *EGF* than those fed with no GOS on d 8 (*P* < 0.05), and there was also a significantly higher mRNA expression of *GCG* in GOS-fed piglets than in those fed with no GOS on d 21 (*P* < 0.05).

The effects of early intervention with GOS on the protein expression of intestinal growth factors are shown in Fig. 3e-h. The results show that the protein expression of GLP-2 and EGF were affected by diet. Piglets fed with diets containing GOS showed significantly higher protein expression of GLP-2 and EGF than those fed with no GOS (*P* < 0.05).

### Disaccharidase activity and nutrient transporters

**Fig. 4:**
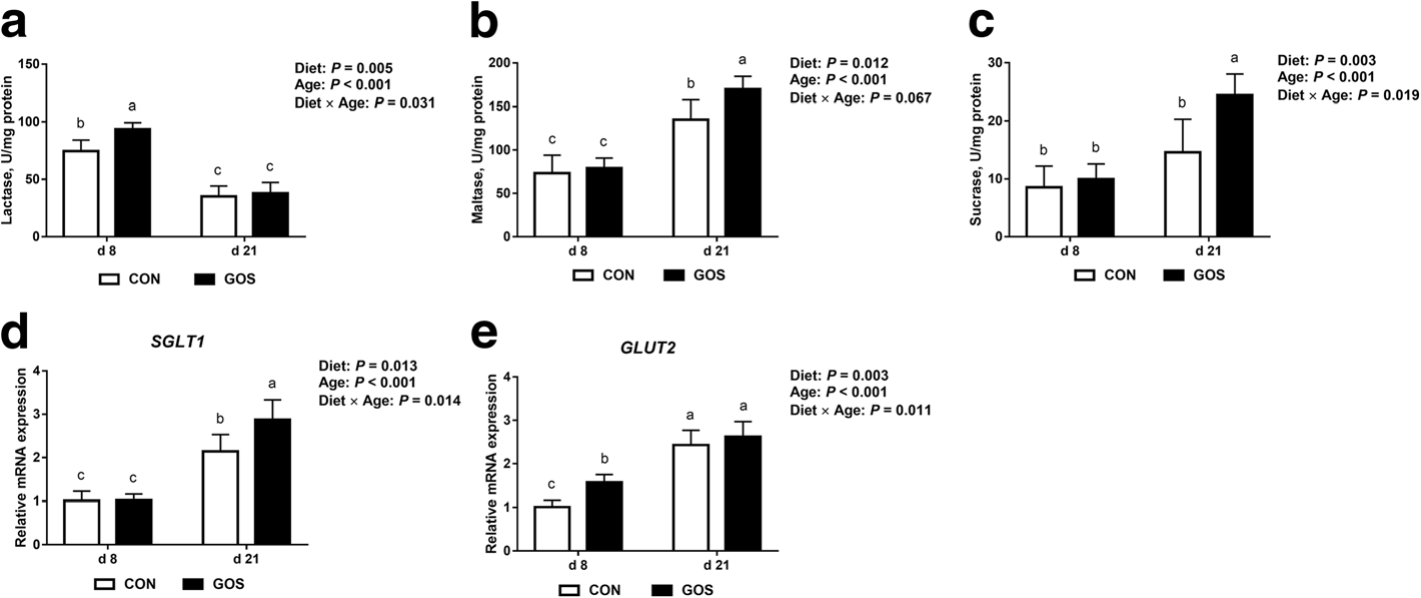
The jejunal disaccharidase activity and mRNA expression of the glucose transport receptors in suckling piglets. Piglets assigned to CON (*n* = 6) and GOS (*n* = 6) received physiological saline and GOS solution for 7 d after birth, respectively. (**a**-**c)** The brush border enzyme activity of the jejuna in suckling piglets. (**d**-**e)** The relative mRNA expression of jejunal nutrient transporter in suckling piglets. The values were calculated relative to the expression of *β-actin* with formula 2^-∆∆Ct^. Values are expressed as means ± SD. Bars assigned with different lower-case letters indicate significant differences. CON: control group; GOS: GOS group

As shown in Fig. 4a-c, lactase and sucrase activity were affected by interactions between diet and age (*P* < 0.05), and maltase activity was affected by diet. More specifically, piglets fed with diets containing GOS showed significantly higher maltase and sucrase activities (*P* < 0.05) than those fed with no GOS on d 21, while piglets fed with diets containing GOS showed significantly higher lactase activity (*P* < 0.05) than those fed with no GOS on d 8.

To assess gene expression related to the nutrient transporters of jejunum, two kinds of genes were assayed by q-PCR, as shown in Fig. 4d-e. It was demonstrated that the mRNA expression of *SGLT1* and *GLUT2* was affected by interactions between diet and age (*P* < 0.05). Piglets fed with diets containing GOS showed significantly higher mRNA expression of *GLUT2* (*P* < 0.05) than those fed with no GOS on d 8, while piglets fed with diets containing GOS showed significantly higher mRNA expression of *SGLT1* (*P* < 0.05) than those fed with no GOS on d 21.

### *D*-lactic acid, diamine oxidase, tight junction genes, and protein expressions

The plasma *D*-lactic acid concentration and DAO activity in the suckling pigs are presented in Table 2. Significant interactions between diet and age (*P* < 0.05) were observed in the plasma *D*-lactic acid concentration, plasma DAO, and jejunal mucosa DAO. On d 8, GOS significantly reduced plasma *D*-lactic acid concentration and DAO activity (*P* < 0.05), and increased jejunal mucosa DAO (*P* < 0.05).

**Table 2:** Effects of galacto-oligosaccharides (GOS) on *D*-lactic acid and diamine oxidase (DAO) in suckling piglets^*a*^.

Items	d 8		d 21		SEM	*P*-value		
	CON	GOS	CON	GOS		Diet	Age	Diet × Age
Plasma *D*-lactic acid, mg/L	13.13 ^a^	12.42 ^b^	12.62 ^b^	12.81 ^ab^	0.15	0.116	0.661	0.010
Plasma DAO, units/mL	3.67 ^a^	3.26 ^b^	3.24 ^b^	3.25 ^b^	0.08	0.019	0.008	0.012
Jejunal mucosa DAO, units/mg mucosa	2.18 ^b^	2.32 ^a^	2.20 ^b^	2.21 ^b^	0.03	0.017	0.170	0.025

^*a*^Piglets assigned to CON (*n* = 6), GOS (*n* = 6) received physiological saline and GOS solution for 7 d after birth, respectively

**Fig. 5:**
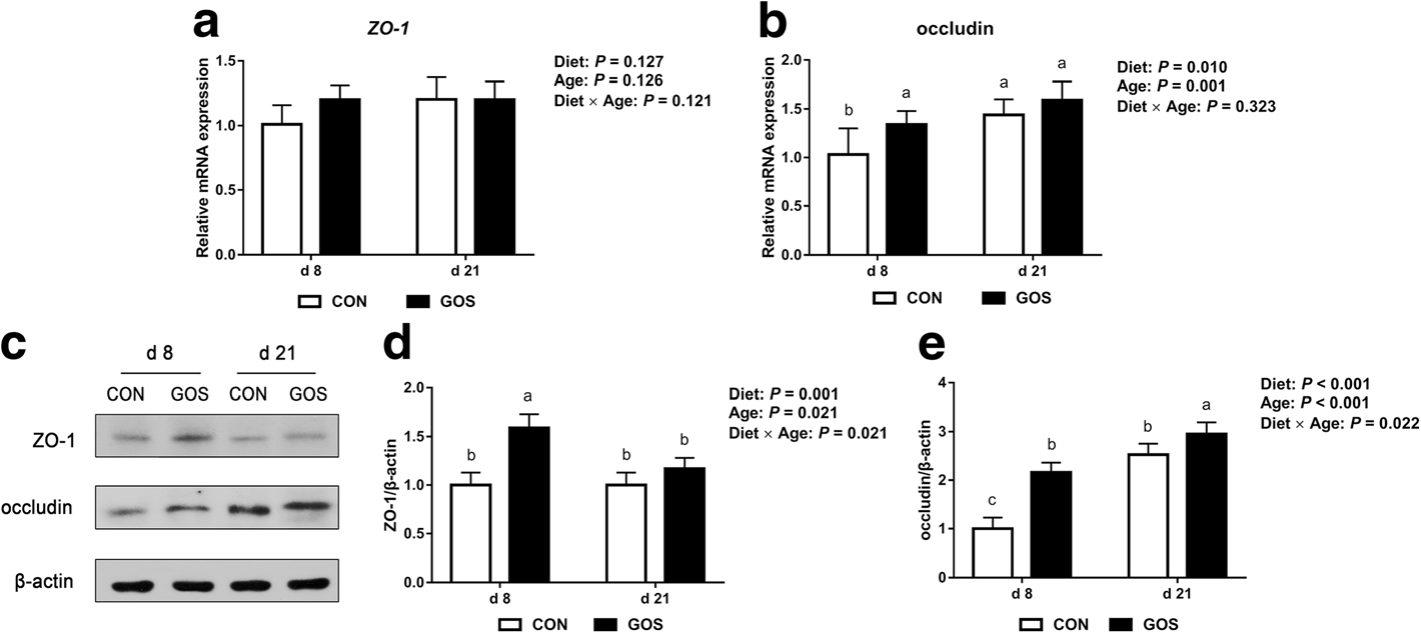
The relative mRNA and protein expression of the jejunal tight junction in suckling piglets. Piglets assigned to CON (*n* = 6) and GOS (*n* = 6) received physiological saline and GOS solution for 7 d after birth, respectively. (**a**-b) The relative mRNA expression of the jejunal tight junction in suckling piglets. The values were calculated relative to the expression of *β-actin* with formula 2^-∆∆Ct^. (**c)** The blots of zonula occludens-1 (ZO-1), occludin, and β-actin of the jejunum mucosa in suckling piglets. (**d**-**e)** The relative protein expressions of the jejunal tight junction in suckling piglets. The value of protein expression was the ratio of the densitometry units of tight junction protein to β-actin. Values are expressed as means ± SD. Bars assigned with different lower-case letters indicate significant differences. CON: control group; GOS: GOS group

Figure 5 shows the gene expression and protein expression of ZO-1 and occludin in the jejunal mucosa of the piglets. Significant dietary effects were observed on the occludin mRNA expression levels (*P* < 0.05), and a higher mRNA expression of occludin was observed in piglets fed with GOS (*P* < 0.05) in contrast to those fed with no GOS on d 8 (Fig. 5a-b). As shown in Fig. 5c-e, the protein expression of ZO-1 and occludin was affected by interactions between diet and age (*P* < 0.05). The protein expression of ZO-1 and occludin in the jejunal mucosa also increased in the GOS group on d 8 (*P* < 0.05). In addition, the protein expression of occludin in the jejunal mucosa of the GOS piglets was higher than that of the CON piglets.

### Intestinal immune factors

**Fig. 6:**
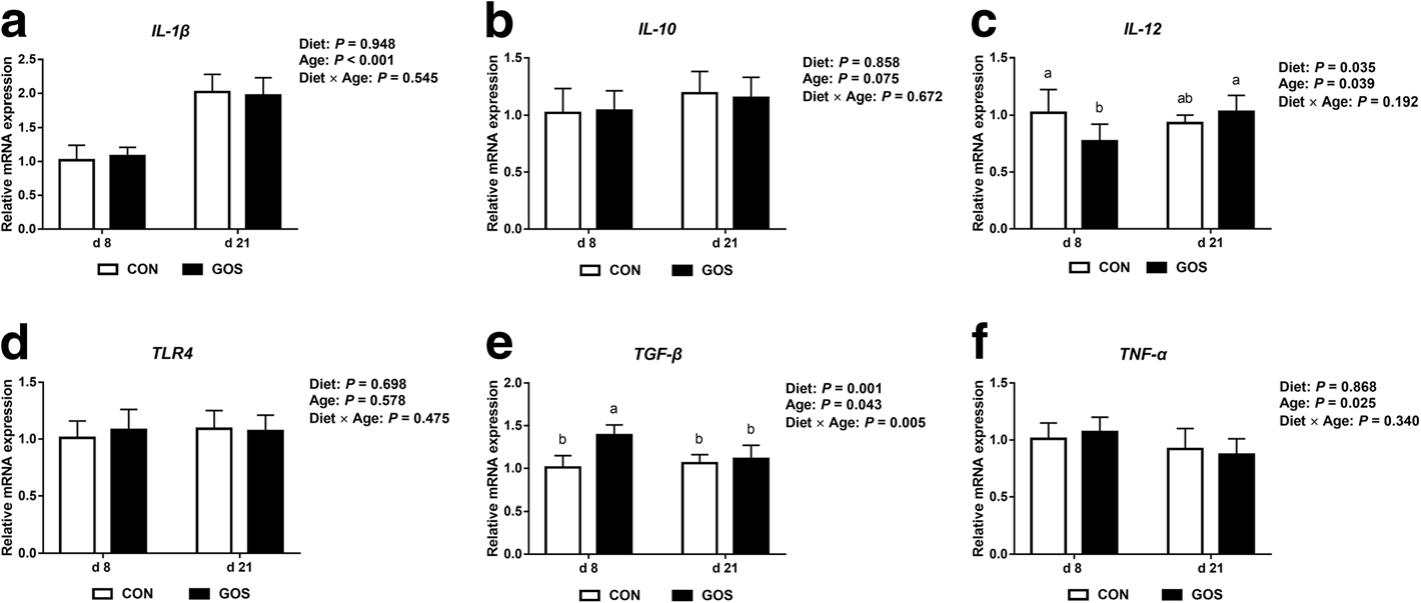
The jejunal immune function in suckling piglets. Piglets assigned to CON (*n* = 6) and GOS (*n* = 6) received physiological saline and GOS solution for 7 d after birth, respectively. (**a**-**f)** The relative mRNA expression of jejunal immune factors in suckling piglets. The values were calculated relative to the expression of *β-actin* with formula 2^-∆∆Ct^. Values are expressed as means ± SD. Bars assigned with different lower-case letters indicate significant differences. CON: control group; GOS: GOS group

With respect to intestinal immune factors (Fig. 6), no significant interactions between diet and age were observed on the mRNA expression of *IL-1β*, *IL-10*, *TLR4* and *TNF-α* (*P* > 0.05). However, significant interactions between diet and age were observed on the *TGF-β* mRNA expression levels (*P* < 0.05), and significant dietary effects were observed on the *IL-12* mRNA expression levels (*P* < 0.05). It was also determined that GOS increased the mRNA expression of *TGF-β* (*P* < 0.05) and reduced the mRNA expression of *IL-12* on d 8 (*P* < 0.05).

## Discussion

In the present study, a neonatal piglet model was used to study the effects of early intervention with GOS on growth performance and jejunal development during a week-long intervention period. By determining the effects of early feeding strategies on the entire suckling stage, the current experiment made it possible to evaluate the effects of GOS (from d 1 to d 7) on piglets’ growth performance, jejunal morphology, disaccharidase activity, and barrier function at different ages. The results suggested that GOS had significant effects on ADG, SI length, crypt depth, disaccharidase activity, tight junction expression, and gut permeability in suckling piglets.

In our study, we referred to the dose of GOS used in rodent model to determine the appropriate dose of GOS for piglets. In previous studies, only the effect of increased abundance of Bifidobacterium has been reported in mice fed GOS of 0.26 g/(kg·d) [10]. While increased cecal total weight and wall weight have been reported in mice fed GOS of 1 g/(kg·d) [16]. Anthony et al. [15] found that rats fed GOS of 2.5 g/(kg·d) or 5 g/(kg·d) decreased food consumption levels. Based on the reported results, the dose of 1 g GOS/kg weight was administered in our study. In addition, it has been reported that the natural oligosaccharide content of sows is approximately in the range of 0.05–0.1 g/dL [22]. According to the Alizadeh’s [23] research, we estimated that the total oligosaccharide intake from the sow milk was about 0.3–0.6 g/d when the piglets received sow milk of 600 mL/d during the 7 d after birth. In our study, piglets eventually intake GOS of 1.37–2.31 g/d (the initial BW of GOS group: 1.52 kg, the BW on d 7 of GOS group: 2.57 kg) with a consideration of the purity of GOS, about 4 times higher than the oligosaccharide intake solely from sow milk by the piglets.

Previous studies have shown that the small intestine has demonstrated a significant increase in tissue mass and surface area of absorption in neonatal piglets [24–28]. For instance, the number of mucosal cells reportedly increased by 50% on the first day after birth and doubled on the third day after birth [28]. These studies indicated that the intestine of piglets developed fastest in the early stages of suckling piglets. Furthermore, it has been reported that GOS could increase cecal total weight and wall weight in mice [16]. Therefore, the purpose of this study was to improve intestinal development and increase the growth performance in the early stages of suckling piglets by supplementing with GOS. Consistent with our purpose, the SI length was significantly increased in GOS group on d 8. The increased SI length indicated that a significant increase in the area of the digestive and absorption of nutritious substances, thereby improving the growth performance of piglets. In addition, there have been several attempts to demonstrate the use of GOS as potential promoters to enhance animal growth [29, 30]. Along the same lines as these studies, we have observed that early intervention with GOS could improve the BW and ADG of suckling piglets, which also consistent with our purpose.

For suckling piglets, intestinal growth factors play a key role in the development of the intestine. For example, the GLP-1, GLP-2, EGF, and IGF-1 proteins were able to increase the proliferation, differentiation, and apoptosis of intestinal epithelial cells [31, 32]. In this study, we observed that the expressions of the intestinal growth factors differed between GOS and CON groups on d 8, but not on d 21. But interestingly, the expressions of intestinal growth factors in GOS piglets on d 8 were close to those in CON piglets and GOS piglets on d 21. According to previous results, the concentrations of growth factors in sow milk at early lactation stage are higher than those at the late lactation stage. This may cause the growth and development of the jejunum to reach the plateau stage at the late period of lactation [33]. And our dynamic change of daily weight gain confirms this speculation. Furthermore, we also observed that the mRNA expression of *IGF-1* and *GCG* (the precursor of glucagon and other components is encoded by the GCG) in the GOS group was higher than that in CON group, and the protein concentration of GLP-2 was consistent with the mRNA expression of the *GCG*. The increased mRNA and protein expression of GLP-2 could increase SI length through the stimulation of epithelial cell antiapoptotic actions by activators of the PI3K-Akt pathway [31]. The activation of Akt in the intestinal mucosa has also been implicated in GLP-2-mediated epithelial glucose uptake [31]. In addition, IGF-1 has been identified as a major mediator through which GLP-2 increases intestinal growth [34]. Also, high mRNA and protein expression of GLP-2 may also be modulated by nutrient intake, especially carbohydrate intake [35, 36]. Therefore, we believe that GOS could improve the growth performance of suckling piglets via promoting jejunal development and increasing carbohydrate intake.

Since the jejunum is the main organ for nutrient absorption, we further analyzed jejunal morphology, disaccharidase activity, and carbohydrate transporters. In the present study, early intervention with GOS significantly decreased the crypt depth on d 21, but it did not affect the villus height and villus height: crypt depth ratio in the jejunum. The most direct factor affecting crypt depth was the change in the proliferation rate of intestinal stem cells [37]. In addition, decreased crypt depth indicated that cell proliferation had decreased in the GOS group. Villus height has been positively correlated with the number of cells present [37]. In this study, there was no difference in the villus height between the two groups, which indicated that decreased cell proliferation did not affect the growth of the jejunum. This may have been caused by the increased cell differentiation and the decreased cell apoptosis of the jejunum. Furthermore, disaccharidase activity is related to intestinal morphology. And the disaccharidase activity determines suckling piglets’ capacity for carbohydrate digestion and transport. In previous studies, lactase activity was high at birth but decreased with the age. However, sucrase activity and maltase activity were low at birth, but their activity gradually increased with the age until reaching stability [38]. Consistent with the findings of these studies, the lactase activity on d 21 was lower than that on d 8, and the sucrase and maltase activities on d 21 were higher than those on d 8. It is known that maltase and sucrase activities are important markers to evaluate intestinal development [39, 40]. Hence, the increase of maltase and sucrase activities implied a certain rapid maturation of the jejunum. In addition, the present study showed that early intervention with GOS up-regulated lactase activity on d 8, and maltase, sucrase activities on d 21. Furthermore, the up-regulated lactase activity on d 8, and the maltase, sucrase activities on d 21 would promote the polysaccharides in sow milk to be degraded into monosaccharides. This is conducive to the body absorbing and utilizing the carbohydrates, thereby promoting intestinal maturity and host growth. These results therefore suggested that the piglets in the GOS group could utilize carbohydrates more efficiently than those in the CON group. After hydrolyzation by disaccharidase, the carbohydrates in a diet depend on a carbohydrate transporter to enter the bloodstream. In this study, the mRNA expressions of *SGLT1* and *GLUT2* were higher in the piglets with GOS intervention than those in the piglets without GOS intervention, indicating an increased glucose transport rate of the intestine. Overall, these results indicate that early intervention with GOS enhances the degradation rate of carbohydrates and the glucose transport rate in suckling piglets by modulating disaccharidase activity and the expression of glucose transport receptors.

A good mechanical barrier can effectively prevent bacteria, endotoxins, and other harmful substances from penetrating the intestinal mucosa, and in terms of infrastructure, it functions as a tight junction between the intact intestinal epithelial cells and other epithelial cells [41–43]. ZO-1 and occludin are main transmembrane and nonmembrane proteins that form intercellular junctions between the epithelial cells [44, 45]. In addition, DAO and *D*-lactate serve as indicators of intestinal integrity, as they are normally presented in very small amounts in blood circulation. Increased plasma *D*-lactic acid levels and serum DAO levels reflect changes in intestinal permeability, suggesting that the intestinal barrier function has been damaged [46, 47]. Many studies have shown that GOS can reduce gut permeability and increase tight junction expression in vivo and in vitro [48, 49]. Consistent with previous research results, our study shows that early intervention with GOS could improve the protein expression of ZO-1 and occludin in the jejunal mucosa on d 8. We also observed that plasma *D*-lactate and DAO decreased in the GOS group on d 8. These results indicated that early intervention with GOS could enhance the barrier function of the jejunum. Furthermore, the improvement of intestinal barrier function may imply the improvement of intestinal immune function. Therefore, we analyzed the mRNA expression of inflammatory factors. In this study, GOS increased the mRNA expression of *TGF-β* and reduced the mRNA expression of *IL-12* on d 8. IL-12 is a pro-inflammatory cytokine, and TGF-β is an anti-inflammatory cytokine. According to the results, early GOS intervention promoted the maturation of immune function. In addition, several studies have shown that the strength of the intestinal barrier was associated with enhanced piglet performance [43, 50]. In our study, the improvement of intestinal barrier function was accompanied by increased growth performance. Therefore, increased barrier function may ensure the absorption of nutrients and prevent bacteria, endotoxins, and other harmful substances from entering the body through the intestinal mucosa [51].

## Conclusion

In conclusion, the results obtained in the present study indicate that the increased in piglet growth with GOS supplementation was associated with the changes in expression of the genes and proteins involved in gut endocrine and barrier function, glucose transporter and immune status. Further study is needed to investigate the exact mechanisms by which GOS can promote intestinal development in suckling piglets.

## Additional file

Additional file 1: **Table S1.** Primer sequences for quantitative real-time PCR analysis. (DOCX 17 kb)

## Abbreviations

ADG: Average daily gain
BCA: Bicinchoninic acid
DAO: Diamine oxidase
DM: Dry matter
DP: Degree of polymerization
EGF: Epidermal growth factor
GAPHH: Glyceraldehyde phosphate dehydrogenase
GCG: Preproglucagon
GLP-2: Glucagon-like peptide-2
GLUT2: Glucose transporter type 2
GOS: Galacto-oligosaccharides
HE: Hematoxylin and eosin
IGF-1: Insulin-like growth factor 1
IGF-1R: Insulin-like growth factor 1 receptor
IL-10: Interleukin-10
IL-12: Interleukin-12
IL-1β: Interleukin-1β
PBS: Phosphate buffer saline
PBST: Phosphate buffer, saline with Tween-2
PVDF: Polyvinylidene difluoride
SDS–PAGE: Sodium dodecyl sulfate–polyacrylamide gel electrophoresis
SGLT1: Sodium glucose co-transporter 1
SI: Small intestine
TGF-β: Transforming growth factor-β
TLR4: Toll-like receptor 4
TNF-α: Tumor necrosis factor-α
ZO-1: Zonula occludens-1
