# Supplementary material for: Effects of galacto-oligosaccharides on growth and gut function of newborn suckling piglets
Source: J Anim Sci Biotechnol. 2018 Oct 18;9:75. doi: 10.1186/s40104-018-0290-9 (PMC6193306; doi:10.1186/s40104-018-0290-9)
Supplement: Supplementary file 1 — Table S1. Primer sequences for quantitative real-time PCR analysis. (DOCX 17 kb) [file 40104_2018_290_MOESM1_ESM.docx]

**Table S1** **Primer sequences for quantitative real-time PCR analysis.**

| Gene^a^ | Nucleotide sequences 5’-3’ | Product size (bp) |  |
| --- | --- | --- | --- |
| *SGLT1* | CCACTTTCCCTATAAAACCTCAC/CTCCATCAAACTTCCATCCTCAG | 151 | NM_001164021.1 |
| *GLUT2* | CCTGCTTGGTCTATCTGCTGTG/TTGATGCTTCTTCCCTTTCTTT | 194 | NM_001097417.1 |
| *IGF-1* | CCTGCGCAATGGAATAAAGTC/GCAAAGTCTGGAAATGAATTGGT | 120 | XM_021091140.1 |
| *IGF-1R* | GGGATGACGAGAGACATCTATGAG/GAAGGACCAGACTCAGAGTGC | 131 | XM_021082920.1 |
| *GCG* | ACTCACAGGGCACGTTTACCA/AGGTCCCTTCAGCATGTCTCT | 150 | XM_005671883.3 |
| *EGF* | ATCTCAGGAATGGGAGTCAACC/TCACTGGAGGATGGAATACAGC | 166 | XM_021100462.1 |
| *ZO-1* | GAGGATGGTCACACCGTGGT/GGAGGATGCTGTTGTCTCGG | 169 | XM_021098896.1 |
| *occludin* | ATGCTTTCTCAGCCAGCGTA/AAGGTTCCATAGCCTCGGTC | 176 | NM_001163647.2 |
| *IL-1β* | AGTGGAGAAGCCGATGAAGA/CATTGCACGTTTCAAGGATG | 113 | XM_021085847.1 |
| *IL-10* | GTCCGACTCAACGAAGAAGG/GCCAGGAAGATCAGGCAATA | 73 | [NM_214041.1](https://www.ncbi.nlm.nih.gov/entrez/viewer.fcgi?db=nucleotide&id=47524185) |
| *IL-12* | TCCTGGGAAAGTCCTGTCGT/GGTGAGGTCGCTAGTTTGGA | 81 | [NM_213993.1](https://www.ncbi.nlm.nih.gov/entrez/viewer.fcgi?db=nucleotide&id=47522811) |
| *TLR4* | TCAGTTCTCACCTTCCTCCTG/GTTCATTCCTCACCCAGTCTTC | 166 | NM_001293316.1 |
| *TGF-β* | GGTGCTAATGGTGGAAAGCG/GGAGCTCCGACGTGTTGAA | 92 | [NM_214015.2](https://www.ncbi.nlm.nih.gov/entrez/viewer.fcgi?db=nucleotide&id=1148291168) |
| *TNF-α* | CCACGCTCTTCTGCCTACTGC/GCTGTCCCTCGGCTTTGAC | 168 | [NM_214022.1](https://www.ncbi.nlm.nih.gov/entrez/viewer.fcgi?db=nucleotide&id=47522865) |
| *GAPDH* | CCAAGGAGTAAGAGCCCCTG/AAGTCAGGAGATGCTCGGTG | 125 | NM_001206359.1 |
| *β-actin* | ATGCTTCTAGACGGACTGCG/ GTTTCAGGAGGCTGGCATGA | 109 | XM_003357928.4 |

*^a^SGLT1*: *Sodium glucose co-transporter 1*; *GLUT2*: *Glucose transporter type 2*; *GCG*: *Preproglucagon*; *IGF-1*: *Insulin-like growth factor 1*; *IGF-1R*: *Insulin-like growth factor 1 receptor*; *EGF*: *Epidermal growth factor*; *ZO-1*: *Zonula occludens-1*; *IL-1β*: *Interleukin-1β*; *IL-10*: *Interleukin-10*; *IL-12*: *Interleukin-12*; *TLR4*: *Toll like receptor 4*; *TGF-β*: *Transforming growth factor-β*; *TNF-α*: *Tumor necrosis factor-α*; Glyceraldehyde phosphate dehydrogenase (*GAPDH*)
